# Microwave-Assisted *Dendropanax morbifera* Extract for Cosmetic Applications

**DOI:** 10.3390/antiox11050998

**Published:** 2022-05-19

**Authors:** Hien Thi Hoang, Jae-Seok Park, Seong-Hyeon Kim, Ju-Young Moon, Young-Chul Lee

**Affiliations:** 1 Department of BioNano Technology, Gachon University, Seongnam-Daero 1342, Sujeong-gu, Seongnam-si 13120, Korea; hienhoangh2t96@gmail.com (H.T.H.); shaera@gachon.ac.kr (S.-H.K.); 2 Nature Fairy Co., Ltd., 3F, 28-27, Dongseo-ro 857 beon-gil, Siheung-si 14983, Korea; ournaturefairy@naver.com; 3 Department of Beauty Design Management, Hansung University, 116 Samseongyoro-16gil, Seoul 02876, Korea; 4 Well Scientific Laboratory Ltd., 305, 3F, Mega-center, SKnTechnopark, 124, Sagimakgol-ro, Jungwon-gu, Seongnam-si 13207, Korea

**Keywords:** *Dendropanax morbifera*, microwave-assisted extraction, cosmetic applications, cosmeceutical ingredient, cytotoxicity

## Abstract

Recently, utilizing natural bioactive compounds for active ingredients in cosmetics has become a growing worldwide trend. More and more studies aim to identify the sources of herbal ingredients for applications in the pharmaceutical and cosmetic fields. Additionally, in order to optimize the safety of natural ingredients, choosing an environmentally friendly extraction method also plays an important role. In this work, an eco-friendly extraction technique for *Dendropanax morbifera* using microwave treatment and microwave-assisted *Dendropanax morbifera* extract (MA-DME) was investigated. The results indicate that higher yields of MA-DME were obtained than with conventional methods and that *D. morbifera*’s antioxidant properties were enhanced. Moreover, we found that MA-DME exhibited extraordinary antioxidant, anti-aging, and skin-whitening activities. We suggest MA-DME as a potential cosmeceutical ingredient than could be utilized for comprehensive protection of human skin.

## 1. Introduction

Not long ago, a global trend toward the use of natural bioactive substances as cosmetic agents took shape, due to their effective and biologically active substances and growing interest in skin care [1]. Currently, the global introduction of plant extracts is focused on applications with high added value, given that plant extracts contain bioactive ingredients including vitamins or minerals. Most of the components are of great interest to the preparation of natural products in cosmetics [2]. The bioactive components from plant extracts can be used as cosmeceutical formulations and achieve benefits including the maintenance of skin structure or function. In order to optimize the safety of natural ingredients, choosing an environmentally friendly extraction method also plays an important role. Microwave-assisted extraction (MAE) has been indicated as a promising technique for the extraction of medicinal plants for research as well as a green approach [3]. MAE provides effective extraction performance with less or no solvent consumption, as well as and protection with rapid and high extraction yields for thermolabile constituents. MAE showed advantages that are more effective and cheaper than conventional extraction methods such as Soxhlet, percolation, extraction under reflux, and sonication [4]. In addition, MAE allowed higher recoveries without altering the antioxidant potential of the extracts [5]. Hence, MAE is one of the most suitable solutions for green and natural products.

*Dendropanax morbifera* (*D. morbifera*), a native plant of South Korea, is known as a medicinal herb used for the comprehensive treatment of human illnesses [6] such as hyperglycemia, hair loss, and skin disorders [7,8,9]. It is reported that many bioactive compounds were are included in *Dendropanax* plant species [10]. Many scientific reports have previously highlighted that the main bioactive compounds of *D. morbifera* are quercetin, chlorogenic acid, rutin, carnosol, dextromethorphan, cannabidiol, bremazocine, doxapram, etc. [11,12]. Furthermore, previous studies reported that *D. morbifera* exhibits antioxidant [13,14], anti-inflammatory [15,16], neuroprotective [6,17], osteosarcoma [18], anti-diabetic [19,20], hepatoprotective [21,22], immunomodulatory [16,23], antibacterial and antifungal [24], antiplasmodial [25], cytotoxic [26], and larvicidal [27] functions. Recently, interest in *D. morbifera* as a functional material in cosmetics has increased thanks to its well-rounded health benefits for humans. There are several reports that *D. morbifera* and its components shows anti-wrinkle, hair growth, and moisturizing effects [7,28]. Especially, *β*-sitosterol, isolated from *D. moribifera*, provides skin whitening and moisturizing effects, prevention of hair loss, and hair density improvement [28]. Moreover, *D. morbifera* leaf extract considerably reduces melanin content, suggesting it as a candidate for a skin-whitening agent [29].

In the present study, we focused on extracting the bioactive compounds in *D. morbifera* by the microwave-assisted method. To further investigate the efficacy of MAE, the active ingredients in microwave-assisted *D. morbifera* extract (MA-DME) were identified by ultra-high performance liquid chromatography with mass spectrometry (UHPLC-MS). In addition, we evaluated the antioxidant, anti-wrinkle, whitening and moisturizing effects to investigate the potential of *D. morbifera* extract as a promising ingredient for cosmetic formulations.

## 2. Materials and Methods

### 2.1. Materials

*D. morbifera* was grown and harvested in Wando, Korea. Methanol and acetonitrile were purchased from Honeywell Burdic & Jackson (Morristown, NJ, USA). Formic acid, sodium carbonate (Na_2_CO_3_), and aluminum chloride hexahydrate (AlCl_3_·6H_2_O) were purchased from Daejung Chemicals & Metals Co., Ltd. (Siheung-si, Korea). EZ-cytox solution was purchased from DoGenBio (Guro-gu, Korea). Tris-HCl buffer, PBS, and Tris solution buffer were purchased from Thermo Fisher Scientific (Waltham, MA, USA). Folin–Denis reagent, kojic acid, elastase, mushroom tyrosinase, 2,2′-azino-bis(3-ethylbenzothiazoline-6-sulfonic acid) (ABTS), Cell Proliferation Kit I (MTT), DMSO, L-ascorbic acid, gallic acid, and quercetin were purchased from Sigma Aldrich (St. Louis, MO, USA)

### 2.2. Microwave-Assisted Dendropanax morbifera Extract (MA-DME)

For the preparation of raw *D. morbifera*, dry leaves and wood of *D. morbifera* were thoroughly soaked in water, then sliced into small pieces. Next, 20 g of the sliced raw materials were ground into a homogenous mixture using a blender. *D. morbifera* powder was obtained by utilizing a freeze-dryer and diluting in various concentrations with DI water. DI water was obtained utilizing Milli-Q Millipore filter system (Millipore Co., Billerica, MA, USA) with conductivity of <18.2 MΩ·cm^2^.

**Figure 1:**
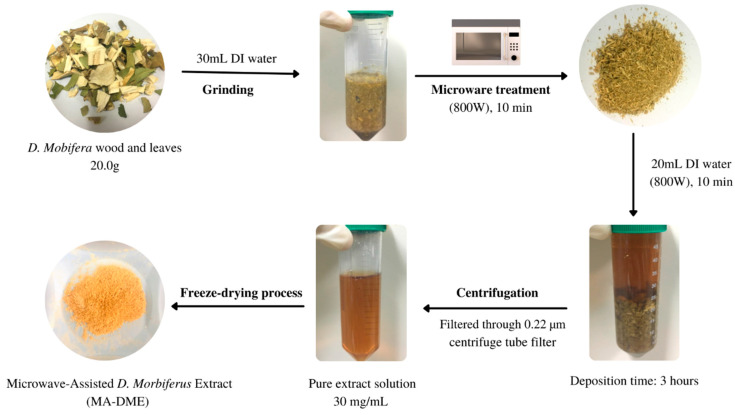
The extraction process for MA-DME.

A microwave oven (Magic MMO-20M7, SK magic, Jongno-gu, Korea) was utilized in this work. The extraction of MA-DME was carried out referring to the method of Alvand et al. (Figure 1) [30,31]. Raw *D.* *morbifera* was irradiated for 10 min under microwave (800 W). The irradiated *D. morbifera* was cooled in ambient temperature and ground into powder. Once the sample was cooled, 20 mL of DI water was added and filtered through 0.22 µm filter. The MA-DME was freeze-dried to obtain extract powder.

The extraction yield Y (%) of MA-DME was expressed in the following equation:

$$Y(\%)=\frac{W_{1}}{W_{2}}\times 100$$

where *W*_1_ is the weight (g) of pure MA-DME powder, while *W*_2_ is the weight (g) of the initial *D. morbifera* powder. Calculated extraction yield was about 33.5%.

To compare the extraction efficiency of MAE with conventional extraction, we prepared Black extract 5% and Transparent extract 5% as control samples. Therein, Black extract 5% was a non-carbonized black extract of *D. morbifera*. After obtaining the pulverized *D. morbifera* particles as in MA-DME, the Black extract was obtained by bathing in water at 100 °C. Additionally, Transparent extract 5% was a non-carbonized transparent extract of *D. morbifera*. After obtaining the pulverized *D. morbifera* particles as in MA-DME, it was subjected to distillation extraction using DI water at 100 °C to obtain a liquid extract through a cooling machine.

### 2.3. Ingredient Analysis of MA-DME

The active compounds of *D. morbifera* were characterized by an ultra-high performance liquid chromatography system loaded with a mass spectrometer (UHPLC-MS) equipped with an electrospray ionization source (ESI). The HPLC separation was conducted on an ACQUITY UPLC HSS T3 column (1.8 µm) with the following parameters: runtime: 25 min; solvent A: formic acid; solvent B: acetonitrile; flow rate: 0.5 mL/min; gradient program: 0 min: 97% A, 0–1 min, 97% A, 1–15 min: 100% B, 15–16 min: 100% B, 16–19 min: 97% A, 19–25 min: 97% A; wavelength: 265 nm; column temperature: 35 °C; sample temperature: 12 °C; injection volume: 5 µL. The mass spectrometer was operated with the following parameters: capillary voltage: 3.0 kV (positive) and 2.8 kV (negative); resolution: 20,000; mass range scanned: 50–1200 m/z; nebulizer pressure: 6.4 Bar; drying gas temperature: 500 °C; drying gas flow rate: 800 L/h.

### 2.4. Antioxidant Content Analysis of MA-DME

Folin–Denis reagent was utilized for measurement of total polyphenol content [32]. A 100 µL amount of MA-DME and 900 µL of DI water were mixed into a conical tube. Then, 100 µL of Folin–Denis reagent were added and incubated at room temperature for 3 min. A 200 µL amount of 10% Na_2_CO_3_ was added after reaction, and the solution was topped up to 2 mL with DI water. The solution was allowed to react for 1 h in the darkroom, and absorbance of the solution was measured at 760 nm using a UV-Vis spectrophotometer (Varian Cary 50 UV-vis spectrophotometer, Agilent Technologies Inc., Santa Clara, CA, USA). The standard calibration curve of gallic acid (GA) was used for calculating total polyphenol content.

Total flavonoid content was also evaluated using modified methods of Woisky and Salatino [33]. In this study, methanolic solutions of quercetin with various concentrations (5–50 µg/mL) were used as references. A 0.6 mL amount of reference solution and 50 mg/mL of MA-DME extract were mixed with 0.6 mL of 2% AlCl_3_. Mixed solutions were incubated at room temperature for 1 h. After incubation, measurement of absorbance was carried out with a UV-Vis spectrophotometer at 420 nm. Determination of total flavonoid content was conducted with quercetin standard curve.

### 2.5. Cell Viability Assay

The effects of MA-DME, Black extract, and Transparent extract on cell viability were evaluated with an MTT assay in 96-well plates. HaCaT cells were seeded at a density of 1⋅10^5^ cells and incubated in 37 °C, 5% CO_2_ conditions for 24 h. After that, various concentration of MA-DME (0 to 300 µg/mL) were treated and incubated for 24 h or 48 h. Then, 10 µL of EZ–cytox solution was added to each well, and the well plate was incubated for 4 h. Finally, measurement of absorbance at 450 nm wavelength was carried out utilizing a plate reader (Multi-label plate reader, PerkinElmer, Boston, MA, USA)**.**

### 2.6. Evaluation of Intracellular Reactive Oxygen Species (ROS)

Dichloro-dihydro-fluorescein diacetate (DCFH-DA) assay was performed to measure the generation of intracellular reactive oxygen species. HaCaT (2.5⋅10^5^ cells/mL) seeded black 96-well plates (Thermo Fisher Scientific, Waltham, MA, USA) were treated with test compounds at a concentration of 0–300 µg/mL and incubated for 24 and 48 h. Then, 10 µM DCFH-DA, which were diluted in DMSO, were allowed to stain for 1 h in a darkroom and washed twice with PBS buffer. In the literature, DCFH-DA penetrates into the internal cells and is then hydrolyzed into DCFH by esters; it was recorded by fluorescence measurement at 485/530 nm immediately. Dipyridamole was used as a control with the relative ROS level of 100%. Generation of ROS was expressed with the following formula:

$$ROS\;Generation(\%)=\frac{F}{F_{0}}\times 100$$

where *F* is the fluorescence intensity of cells pretreated with MA-DME; *F*_0_ is the fluorescence intensity of dipyridamole.

### 2.7. ABTS Radical Preparation Protocol

The antioxidant capacities were measured by ABTS assay using a recent method with a slight modification [34]. The Black extract and MA-DME were suspended in dimethyl sulfoxide (DMSO), and the DMSO alone was used as a negative control. L-Ascorbic acid (Vitamin C) and quercetin were used as antioxidant standards and diluted with DMSO at 10 mg/mL. After 50 µL/well of test compounds were added in the 96-well plate, 150 µL of the ABTS radical was added into each well. Then, absorbance was read at 734 nm at room temperature for 5, 15, and 30 min of incubation time, and initial absorbance was 0.7 (CLARIOstar Microplate Reader, BMG Labtech Inc., Cary, NC, USA). Radical scavenging activity of test compounds was expressed as inhibition of absorbance (*I_a_*, %) using the following formula:

$$I_{a}(\%)=100-[\frac{uExtract\;absorbance}{\mu DMSO\;absorbance}\times 100]$$

where µExtract absorbance is the absorbance of test compounds, µDMSO is the absorbance of the DMSO.

### 2.8. Tyrosinase Inhibitory Activity Assay

The tyrosinase inhibitory activity was measured with reference to a previously reported method [35]. L-DOPA was used as substrate in this assay. A 40 µL amount of 10 mM L-DOPA was mixed with 80 µL of phosphate buffer (0.1 M, pH 6.8) in a 96-well plate and incubated for 10 min at 37 °C. A 40 µL amount of MA-DME or Black or Transparent extract (50, 100, 200, 400, 800, and 1000 µg/mL) and 40 µL of mushroom tyrosinase (250 U/mL, in PBS) were then added to each well. Absorbance of the mixtures was read at 475 nm utilizing a microplate reader (Multi-label plate reader, PerkinElmer, Boston, MA, USA) at 1 min intervals over a period of 120 min. PBS was used as a blank control and Kojic acid (50 µg/mL) and L-ascorbic acid (50 µg/mL) were used as positive controls. The inhibition for each enzyme assay was expressed as follows:

$$Inhibition(\%)=[\frac{control\;OD-Sample\;OD}{Control\;OD}]\times 100$$

where Control OD indicates the difference in absorbance of control between incubation times, and Sample OD indicates the difference in absorbance of sample between incubation times.

Each experiment was performed in triplicate (*n* = 3), and the IC_50_ value was calculated from the dose–response curves by nonlinear regression analysis using GraphPad Prism software version 5.0 (GraphPad software Inc., San Diego, CA, USA).

### 2.9. Elastase Inhibitory Effect Assay

Assessment of elastase inhibition was conducted by the intensity of the solution color assay referring to the method of Tu and Tawata [36]. N-Succinyl-Ala-Ala-Ala-ρ-nitroanilide (AAAVN) elastase substrate was diluted with 0.1232 M Tris-HCl buffer solution (pH 8) to make a 1.0 mM concentration. Then, the elastase substrate was mixed with the 10 µL of sample in the 96-well plates and pre-incubated at 25 °C for 10 min. After pre-incubation, the reaction was initiated by adding 10 µL of elastase from porcine pancreas (7.5 units/mL) in Tris solution buffer to the pre-incubated mixtures. Finally, a microplate reader (Multi-label plate reader, PerkinElmer, Boston, MA, USA) was utilized to measure the absorbance at 410 nm.

### 2.10. Blue Light Penetration Experiment with MA-DME

MA-DME was prepared by dilution at concentrations of 3.75, 7.5, 15, and 30 mg/mL. The 400–450 nm wavelength band of blue light was tested for blocking or transmission with DI water, as control sample, by using a black night UV lamp (ROBUST UV 12LED, Campnic, Uijeongbu-si, Korea).

## 3. Results and Discussion

### 3.1. Characterization of MA-DME

**Figure 2:**
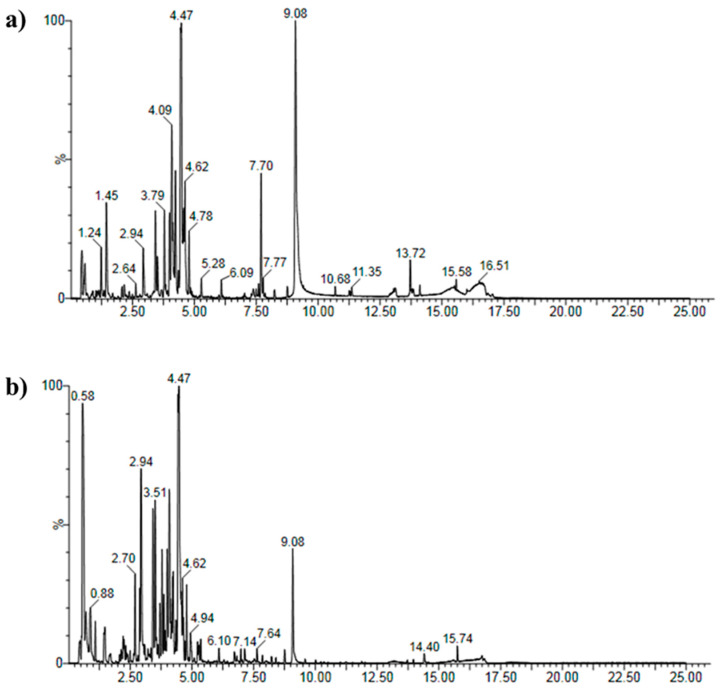
High-resolution extracted ion chromatograms for MA-DME: (**a**) positive ion mode; (**b**) negative ion mode.

The UHPLC-MS chromatogram identified 33 major compounds in *D. morbifera* extract (Figure 2, Table 1). As listed in Table 1, we found that *D. morbifera* extract contains many phenolic and flavonoid compounds known as natural antioxidants, such as 1-*O*-Caffeoylquinic acid, 4-*O*-Caffeoylquinic acid, and Corchoionoside, 3,7,8,3′,4′-Pentahydroxyflavone, whereas apocynoside I inhibits CYP2C9, which causes aging and lowers disease resistance, and Viscumneoside III inhibits tyrosinase, which impedes skin whitening [37].

Quercetin is known to inhibit MMP-1, which encourages the imbalance or collapse of collagen molecules in UV-irradiated skin [38].

### 3.2. Total Contents of Phenols and Flavonoids

Total amounts of phenols were evaluated by modified Folin–Ciocalteu method and expressed as gallic acid equivalents per μg dry weight of plant extract (GAE μg^−1^ DW). A calibration curve for quantification of phenolic compounds in samples was prepared using gallic acid at a concentration of 50 to 500 μg/mL. The calibration curve was formed as a graph of y = 0.02992 + 0.00112x (R^2^ = 0.9969) (Figure S1). The absorbance measurement results are shown in Figure S2 and Table S1. From the above results, it was found that the phenolic compounds were contained in an amount of 313.03 GAE μg^−1^ DW in MA-DME, 252.25 GAE μg^−1^ DW in Black extract 5%, and 30.57 GAE μg^−1^ DW in Transparent extract 5% (Table 2).

In addition, the concentrations of total flavonoids in *D. morbifera* were determined by the modified Woisky and Salatino method and expressed in quercetin equivalents per μg dry weight of plant extract (QE μg^−1^ DW). The calibration curve was formed as a graph of y = 0.005 + 0.0298x (R^2^ = 0.9841) (Figure S3). The absorbance measurement results are shown in Figure S4 and Table S2. From the above results, it was found that flavonoids were contained in an amount of 32.37 QE μg^−1^ DW in MA-DME, 21.04 QE μg^−1^ DW in Black extract 5%, and 0 QE μg^−1^ DW in Transparent extract 5% (Table 2).

In general, the extract of MA-DME was found to have very high contents of both phenolics and flavonoids, compared to the contents in Black and Transparent extracts. It could be explained that microwave treatment heats the material toward its volume while the conventional heating process heats from the outside of the material and requires contact with a hot outer surface. Thus, internal change within a short time leads to pressure increase inside the plant cells, which further breaks the cell walls and releases the desired molecules.

### 3.3. Cell Viability Assay

**Figure 3:**
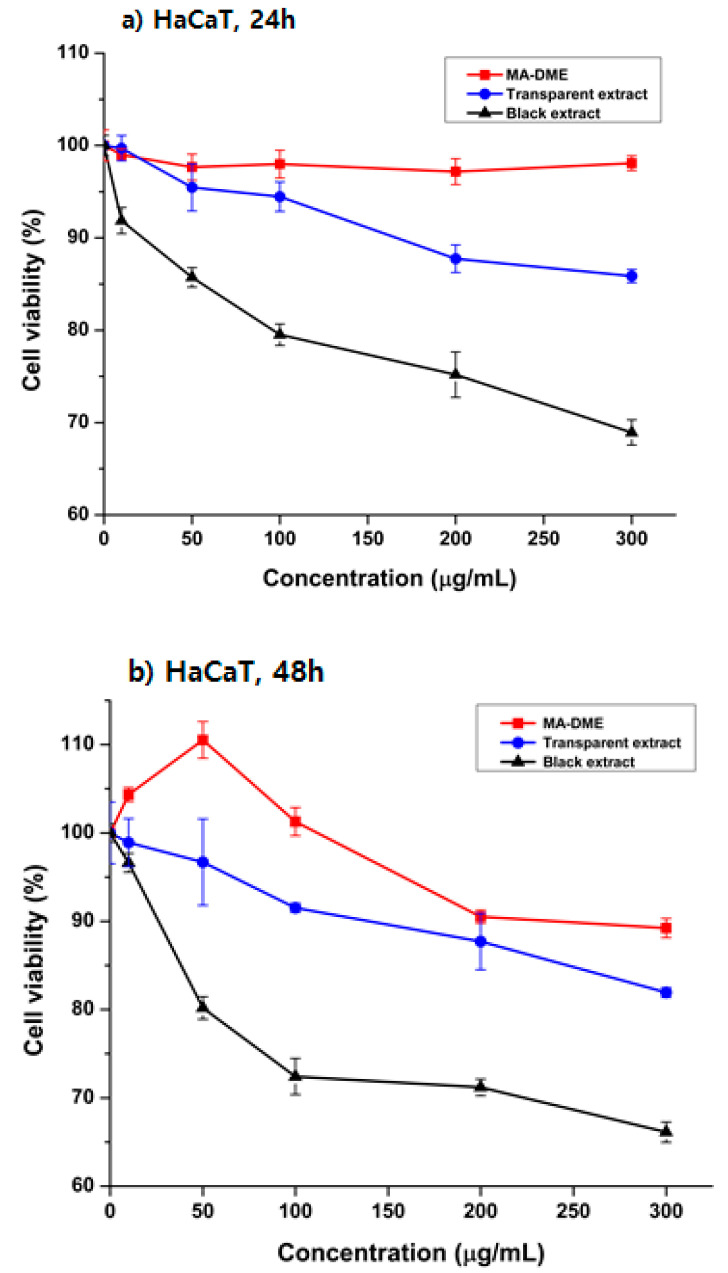
The effect of MA-DME on the viability of HaCaT cells compared with Black extract and Transparent extract for (**a)** 24 h, (**b**) 48 h. The viability of MA-DME (10 to 300 µg/mL) was measured via MTT assay, *n* = 3.

MA-DME did not reduce HaCaT cell viability in concentrations of up to 100 μg/mL, both after 24 h and 48 h, but slightly decreased viability at 200 μg/mL after 48 h (Figure 3). These results determined the survival of human keratinocyte HaCaT cells with treatment of MA-DME in various concentrations (10 to 300 μg/mL). Transparent extract was non-toxic to HaCaT cells at low concentrations (≤200 μg/mL) and caused negligible toxicity at higher concentrations (300 μg/mL).

### 3.4. MA-DME Effect on Reactive Oxygen Species (ROS)

**Figure 4:**
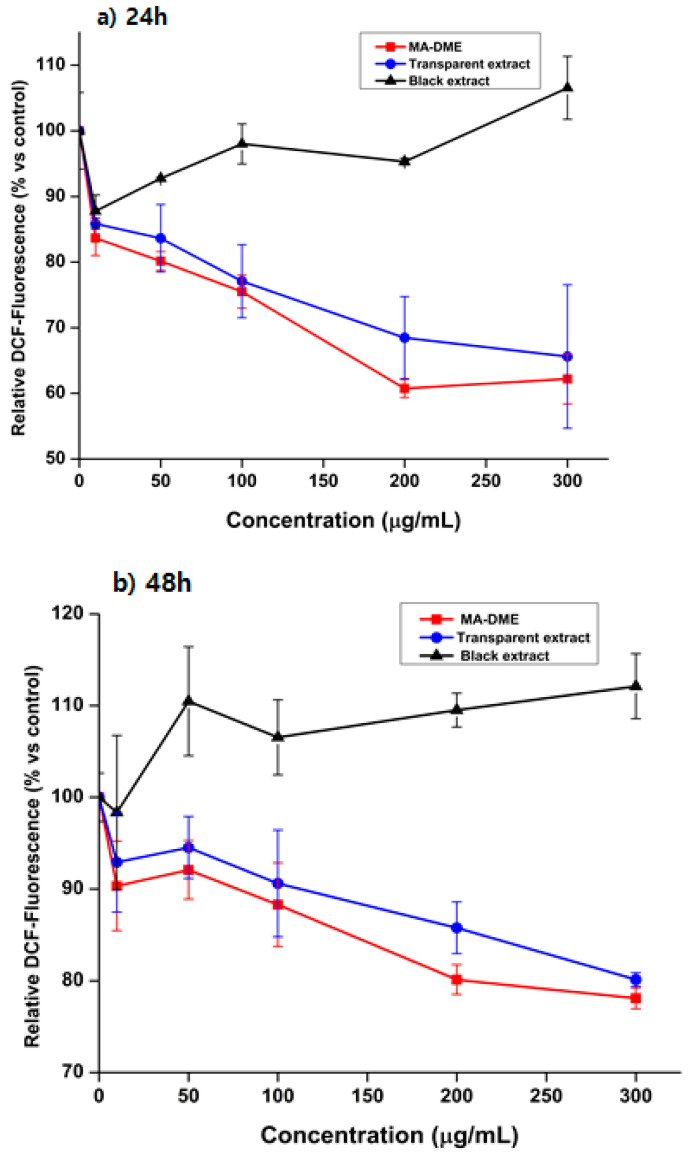
The effect of MA-DME on ROS compared with Black extract and Transparent extract for (**a**) 24 h, (**b**) 48 h , *n* = 3.

The ROS measurements demonstrated that the Transparent extract does not produce ROS radicals in either short or long-term treatment, which may explain that this extract contained a large amount of anti-oxidant compounds. Although the Black extract showed slightly higher toxicity than the Transparent extract and MA-DME, it could be considered to be less toxic to HaCaT cells because more than 60% of the cells could survive at its high concentration (300 g/mL) (Figure 4). Furthermore, this extract also produced a small amount of ROS radicals, resulting in increases in relative DCF-fluorescence to control. In summary, MA-DME, rather than the Black extract and Transparent extract, could be possibly applied to cosmetics.

### 3.5. ABTS Free Radical Scavenging Activity

**Figure 5:**
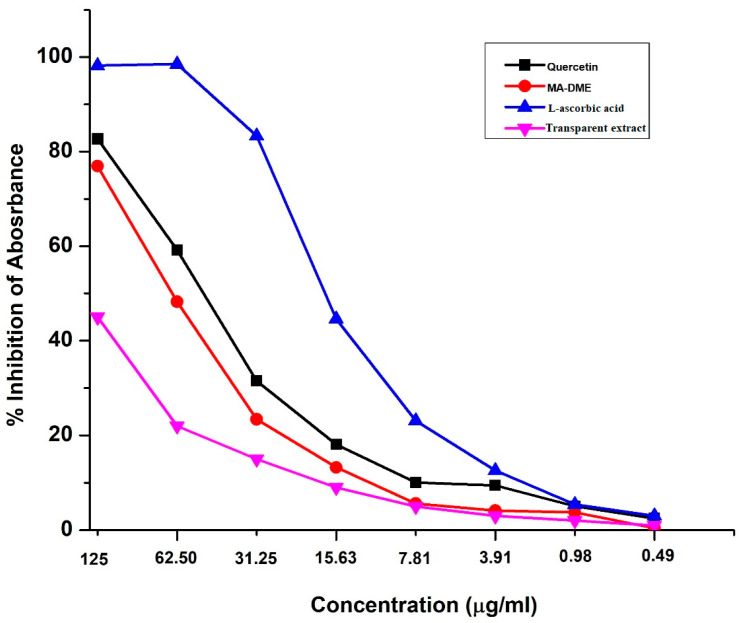
ABTS radical scavenging activity of MA-DME and Transparent extract.

The radical scavenging activities of MA-DME and Transparent extracts were determined by their ABTS radical scavenging efficiency compared to L-ascorbic acid and quercetin (Figure 5). Radical scavenging activities of the MA-DME and Transparent extracts showed concentration-dependent relationships. MA-DME exhibited slightly lower ABTS radical scavenging activities than L-ascorbic acid and quercetin did. On the other hand, the radical scavenging activities of the Transparent extracts were very low, compared with MA-DME, and Transparent extracts only showed a slight concentration-dependent increase in activity.

Extracellular matrix (ECM), the outermost skin part, consists of fibroblasts and protein including collagen and elastin [39]. Collagen and elastin are essential for maintaining skin richness and elasticity to keep it youthful and healthy [40,41]. Deposited ROS due to exposure to photo-aging factors can indirectly activate dermal enzymes such as collagenase and elastase, which basically break down and degrade collagen as well as elastin, respectively [42]. Additionally, external oxidative attacking factors influence the skin and have to cope with the endogenous generation of ROS and other free radicals, which are produced continuously during cellular metabolism. Therefore, MA-DME with its high radical scavenging activities and ROS inhibition can be useful to prevent skin aging.

### 3.6. Tyrosinase Inhibitory Activity Assay

Deposits of melanin in the epidermal layer can cause undesired melanogenesis or skin pigmentation [43]. Melanogenesis could be regulated by inhibiting the activity of tyrosinase or other related enzymes. Tyrosinase, one of the melanogenic enzymes, is the rate-limiting enzyme that controls the production of melanin [44]. Thus, utilizing tyrosinase inhibitors is clearly a promising way to inhibit melanogenesis.

**Figure 6:**
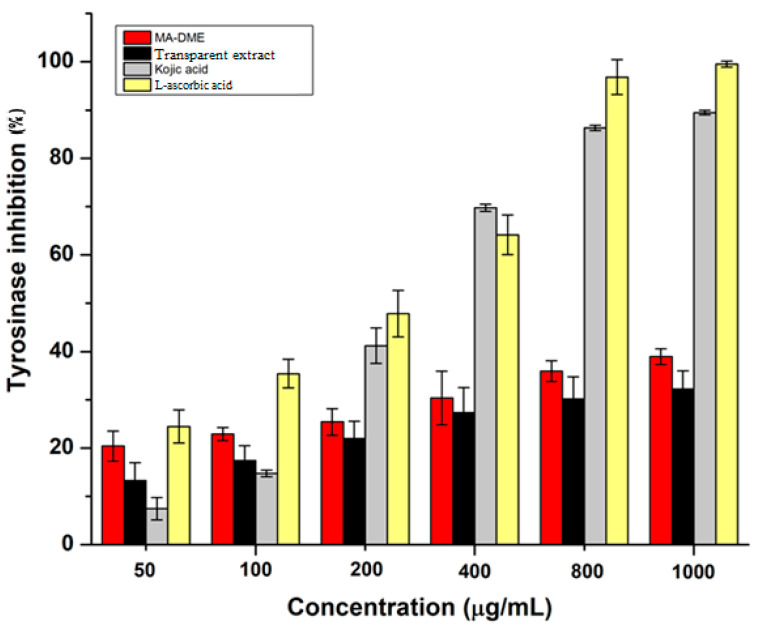
Tyrosinase inhibitory effects of MA-DME, Transparent extract and positive controls, *n* = 3.

The potential of MA-DME to inhibit mushroom tyrosinase at concentrations from 50 to 1000 µg/mL was higher than that of Transparent extract (Figure 6). At concentrations of 50 and 100 µg/mL, MA-DME showed better tyrosinase inhibition effects than kojic acid, which is known as a whitening agent. Consequently, MA-DME is a promising whitening ingredient for cosmetics applications.

### 3.7. Elastase Inhibitory Assay

Elastase is particularly responsible for the disruption of elastin, and elastin is a crucial protein present in the ECM. Elastin, due to its unique elastic recoil properties, is a crucial protein for imbuing elasticity to the skin [45,46]. With regard to anti-aging, seeking to inhibit elastase enzymes is valuable to avoid skin aging and the loss of skin elasticity.

To examine the effects of MA-DME on elastase effects, we determined the porcine elastase activity upon treatment with various concentrations of sample extract (Table 3).

$$*Elastase\;Inhibition\;Ratio(\%)=1-\frac{Absorbance\;of\;sample}{Absorbance\;of\;control}\times 100$$

### 3.8. Blue Light Penetration Experiment with MA-DME

**Figure 7:**
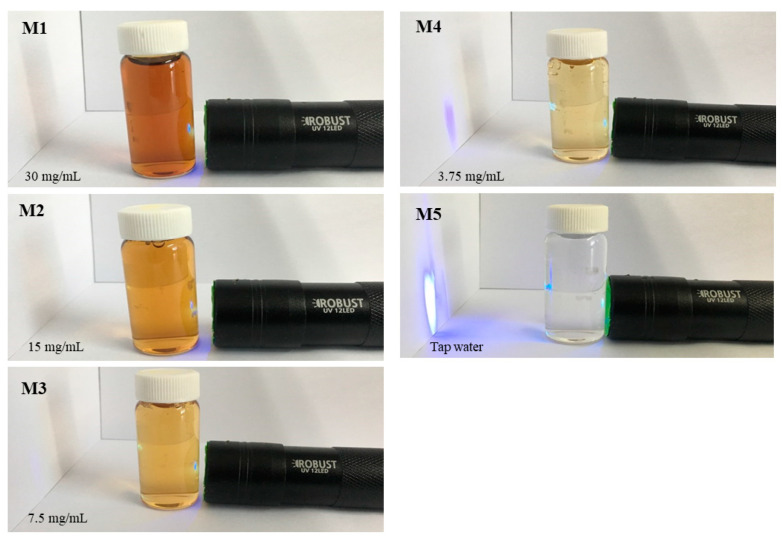
Results for blue light protection effects of MA-DME.

The experimental results are shown in Figure 7. MA-DME did not transmit blue light at concentrations of 30 mg/mL, 15 mg/mL, and 7.5 mg/mL. It was found that the blue light was faintly transmitted in a sample at 3.75 mg/mL concentration. As such, MA-DME was shown to have a shielding ability against blue light. Therefore, MA-DME may have a skin-protecting effect against blue light.

## 4. Conclusions

The results of this study showed that the microwave-assisted extraction method improved the bioactivities of *D. morbifera* in the resultant MA-DME. It was also found that MA-DME showed high biocompatibility, excellent antioxidant activity and whitening effects. Compared with Transparent extract or Black extract, the superior features of MA-DME including cell viability, total bioactive compound contents, radical scavenging effects, and tyrosinase inhibitory effects were shown in this work. Therefore, we suggest MA-DME as a promising candidate for cosmetic applications.

## Figures and Tables

**Table 1:** Identified compounds in MA-DME.

No	Compound	Formula	Ion Mode	Retention Time	Extract Mass (m/z)	Suggested Role
1	Quinic acid	C_7_H_12_O_6_	–	0.59	191.0558	Astringent, anti-viral
2	**1-O-Caffeoylquinic acid**	**C_16_H_18_O_9_**	**–**	**2.95**	**353.0874**	**Phenolic acid, antioxidant, antibacterial**, anticancer, antihistamine, anti-viral
3	4-O-Caffeoylquinic acid	C_16_H_18_O_9_	–	3.52	353.0871	
4	**3-[(4-O-Acetyl-6-deoxy-alpha-L-mannopyranosyl)-oxyl]-2-(3,4-dihydroxyphenyl)-5-hydroxy-6-methoxy-4-oxo-4H-chromen-7-yl 2-O-acetyl-6-deoxy-alpha L-mannopyranoside**	**C_32_H_36_O_18_**	**–**	**3.43**	**707.1825**	**Flavonoids**
5	Benzyl alcohol xylopyranosyl(1->6)glucopyranoside	C_18_H_26_O_10_	–	3.71	401.1437	Tea aroma glycosidic precursor bioactivation
**6**	**Apigenin-6-C-glucosylglucoside (Isovitexin)**	**C_27_H_30_O_15_**	–	**3.79**	**593.1497**	**Anti-inflammatory, antioxidant, antibacterial**, anti-Alzheimer’s disease, anti-diabetic, anti-viral
			+	3.79	595.2663	
7	**Apocynoside I**	**C_19_H_30_O_8_**	–	**3.86**	**431.1901**	**Inhibition of CYP2C9—causing aging and lowered disease resistance**
8	**Corchoionoside C**	**C_19_H_30_O_8_**	–	**3.86**	**431.1909**	**Flavonoids**
9	1,3-Dihydroxy-2-hydromethylanthraquinone-3-B-β-D-xylopyranose(1->6)-β-D-glucopyranoside	C_16_H_28_O_14_	–	4	563.1395	N/A
			+	3.99	565.2557	
10	Apiin	C_16_H_28_O_14_	–	4.09	563.1402	Anxiolytic, **anti-inflammatory**, anti-cancer, anti-fungal
			+	4.09	565.1562	
11	Isochaftoside	C_16_H_28_O_14_	–	4.23	563.1399	Flavones
***12***	***7-O-β-D-gluocopyranosylkaempferol***	***C_21_H_20_O_11_***	**–**	***4.24***	***447.0924***	***Anti-inflammatory*** *, anti-cancer, anti-diabetes* *Inhibit vascular endothelial* ***inflammation*** *Protect the cranial nerve and heart function* *Treat fibroproliferative disorders, anti-viral*
			**+**	**4.13**	**449.1083**	
***13***	***Kaempferol-3,7-di-O-β*** ***-D-glucopyranoside***	***C_27_H_30_O_16_***	**–**	***4.46***	***609.1453***	
			***+***	***4.46***	***611.1618***	
14	Nelumboroside A	C_27_H_30_O_16_	–	4.57	609.1450	Antioxidant
15	3,8-Di-C-glucosylapigenin	C_27_H_30_O_15_	–	4.79	593.1499	N/A
***16***	***Genistein-7,4′-di-O-β*** ***-D-glucoside***	***C_27_H_30_O_15_***	**–**	***4.79***	***593.1499***	*Prevents hypertension, anti-cancer, maintaining bone mineral density, anti-Alzheimer’s disease, anti-viral*
17	Terestigmine	C_21_H_33_N_3_O_3_	–	9.08	374.2436	Cholinesterase inhibitor (treatment of cognition disorders)
18	N-(3-Methoxy-5-nitrophenyl)-2-(5-methyl-3,4-dinitro-1H-pyrazol-1-yl)acetamide	C_13_H_12_N_6_O_8_	+	0.56	381.0795	N/A
19	Guanine	C_5_H_5_N_5_O	+	1.45	152.0566	Nucleobases
20	Daidzein-4′,7-diglucoside	C_27_H_30_O_14_	+	4.42	579.1728	Phytoestrogen
21	Viscidulin I	C15H10O7	+	4.46	303.0502	Inhibitor of hepatocellular carcinoma cells (protein Glypican-3)
22	Viscumneoside III	C_25_H_26_O_13_	+	4.47	535.1454	**Tyrosinase inhibition (skin whitening)**
***23***	*Aloe emodin 8-glucoside*	*C_21_H_20_O_10_*	–	*4.51*	*431.0971*	*Anti-diabetic, DNA targeting molecule, anti-viral*
			*+*	*4.51*	*433.1132*	
24	**3,7,8,3′,4′-Pentahydroxyflavone** **(Quercetin)**	**C_15_H_10_O_7_**	**+**	**4.63**	**303.0499**	**Strong antioxidant flavonoids, xanthine oxidase inhibition, antihyperuricemic, anti-inflammatory, enhances immune-regulation**
25	Rubianic acid (dithiooxamide)	C_25_H_26_O_13_	+	4.65	535.1451	Chelating agent (detection of copper), building block in the synthesis of cyclen
***26***	***3-Hydroxy baicalein***	***C_15_H_10_O_6_***	***+***	***4.79***	***287.0548***	*Anxiolytic, antiestrogen, **anti-inflammatory, anti-cancer, antibacterial**, anti-viral*
27	7-Hydroxy-1-methoxy-2-methoxyxanthone	C_15_H_10_O_6_	+	4.79	287.0548	N/A
28	6,6′-Iminobis(2,2-dimethyl-1-hexanol)	C_16_H_35_NO_2_	+	7.7	274.2738	N/A
29	N~2~-[(2S,4S,5S)-5-Amino-6-cyclohexyl-4-hydroxy-2-isopropylhexanoyl]-N-[2-pyridinylmethyl)-L-isoleucinamide	C_27_H_46_N_4_O_3_	+	9.08	475.3646	N/A
30	4-N-([1,2,4]triazolo[4,3-a]pyridin-3-ylmethyl)butanamide	C_19_H_27_N_9_O	+	9.08	398.2415	N/A
31	1-Methyl-2-[(Z)-8-tetradecenyl]-4(1H)-quinolone	C_24_H_35_NO	+	9.08	276.2596	N/A
32	O-Benzyl-N-[9-(1H-imidazol-1-yl)nonanoyl]-L-seryl-N~6~-[(benzyloxy)carbonyl]-N-(2-cyclohexylethyl)-L-lysinamide)	C_44_H_64_N_6_O_6_	+	9.08	773.4956	N/A

Bold: Main phenolic and flavonoid compounds. Italic and Bold: Potential cosmetic compounds.

**Table 2:** Total phenolic content and flavonoid content in MA-DME.

Sample	Total Phenols (mg GAE g^−1^)	Total Flavonoids (mg QE g^−1^)
MA-DME	313.03 ± 3.9	32.37 ± 0.9
Black extract 5%	252.25 ± 6.9	21.04 ± 1.1
Transparent extract 5%	30.59 ± 4.2	0

**Table 3:** Inhibition activity (%) of MA-DME targeting porcine pancreas elastase activity (0.4 U/mL).

Concentration (µg/mL)	Elastase Inhibition Ratio (%) *		
	MA-DME	Retinol	Adenosine
6.25	4.6 ± 2.0	4.6 ± 2.5	3.5 ± 1.4
12.5	6.8 ± 2.4	13.8 ± 1.0	12.2 ± 0.88
25	13.1 ± 1.2	9.04 ± 3.0	11.8 ± 2.9
50	14.9 ± 1.7	13.6 ± 0.90	14.1 ± 1.0
100	14.1 ± 0.60	13.3 ± 0.21	8.7 ± 1.3
200	15.8 ± 0.61	11.0 ± 1.3	13.0 ± 1.5
400	16.0 ± 0.43	14.5 ± 0.70	13.7 ± 1.6

(Mean ± SD) MA-DME showed high elastase inhibition activity in a specific concentration range. Additionally, MA-DME at high concentrations (100 to 400 µg/mL) showed enhanced active elastase inhibition activity compared to the effect of retinol and adenosine. Therefore, MA-DME might have a potential role in improving skin elasticity and reducing wrinkles in skin.

## Data Availability

Data is contained within the article and supplementary material.

## Supplementary Materials

The following supporting information can be downloaded at: https://www.mdpi.com/article/10.3390/antiox11050998/s1, Table S1. List of samples and the absorbance measurement results for evaluating phenolic; Table S2. List of samples and the absorbance measurement results for evaluating flavonoid; Figure S1. The calibration curve for quantification of phenolic compounds; Figure S2. The absorbance measurement results to evaluate the phenolic compounds; Figure S3. The calibration curve for quantification of flavonoid compounds; Figure S4. The absorbance measurement results to evaluate the flavonoid compounds.

## References

[1] Amberg N., Fogarassy C. (2019). Green Consumer Behavior in the Cosmetics Market. Resources.

[2] Atanasov A.G., Waltenberger B., Pferschy-Wenzig E.-M., Linder T., Wawrosch C., Uhrin P., Temml V., Wang L., Schwaiger S., Heiss E.H. (2015). Discovery and Resupply of Pharmacologically Active Plant-Derived Natural Products: A Review. Biotechnol. Adv..

[3] Mandal V., Mohan Y., Hemalatha S. (2007). Microwave Assisted Extraction—An Innovative and Promising Extraction Tool for Medicinal Plant Research. Pharmacogn. Rev..

[4] Vinatoru M., Mason T.J., Calinescu I. (2017). Ultrasonically Assisted Extraction (UAE) and Microwave Assisted Extraction (MAE) of Functional Compounds from Plant Materials. TrAC Trends Anal. Chem..

[5] Delazar A., Nahar L., Hamedeyazdan S., Sarker S.D. (2012). Microwave-Assisted Extraction in Natural Products Isolation. Nat. Prod. Isol..

[6] Park S.-Y., Karthivashan G., Ko H.M., Cho D.-Y., Kim J., Cho D.J., Ganesan P., Su-Kim I., Choi D.-K. (2018). Aqueous Extract of *Dendropanax morbiferus* Leaves Effectively Alleviated Neuroinflammation and Behavioral Impediments in Mptp-Induced Parkinson’s Mouse Model. Oxidative Med. Cell. Longev..

[7] Park Y.M., Han J.S. (2006). A Study on the Utilization of *Dendropanax morbifera* Lev. Leaf Extract for Material of Functional Cosmetics and Hair Growth Products. Asian J. Beauty Cosmetol..

[8] Kim J.M., Park S.K., Guo T.J., Kang J.Y., Ha J.S., Lee D.S., Lee U., Heo H.J. (2016). Anti-Amnesic Effect of *Dendropanax morbifera* via Jnk Signaling Pathway on Cognitive Dysfunction in High-Fat Diet-Induced Diabetic Mice. Behav. Brain Res..

[9] Kim W., Kim D.W., Yoo D.Y., Jung H.Y., Nam S.M., Kim J.W., Hong S.-M., Kim D.-W., Choi J.H., Moon S.M. (2014). *Dendropanax morbifera* Léveille Extract Facilitates Cadmium Excretion and Prevents Oxidative Damage in the Hippocampus by Increasing Antioxidant Levels in Cadmium-Exposed Rats. BMC Complement. Altern. Med..

[10] Balakrishnan R., Cho D.Y., Su-Kim I., Choi D.K. (2020). *Dendropanax morbifera* and Other Species from the Genus *Dendropanax*: Therapeutic Potential of Its Traditional Uses, Phytochemistry, and Pharmacology. Antioxidants.

[11] Kim J.Y., Yoon J.-Y., Sugiura Y., Lee S.-K., Park J.-D., Song G.-J., Yang H.-J. (2019). *Dendropanax morbiferus* Leaf Extract Facilitates Oligodendrocyte Development. R. Soc. Open Sci..

[12] Yang H.Y., Kim K.S., Lee Y.H., Park J.H., Kim J.-H., Lee S.-Y., Kim Y.-M., Kim I.S., Kacew S., Lee B.M. (2019). *Dendropanax morbifera* Ameliorates Thioacetamide-Induced Hepatic Fibrosis Via Tgf-Β1/Smads Pathways. Int. J. Biol. Sci..

[13] Choi J., Kim S. (2019). Antioxidant and Antithrombotic Properties of *Dendropanax morbifera* Léveille (Araliaceae) and Its Ferments Produced by Fermentation Processing. J. Food Biochem..

[14] Youn J.S., Kim Y.-J., Na H.J., Jung H.R., Song C.K., Kang S.Y., Kim J.Y. (2019). Antioxidant Activity and Contents of Leaf Extracts Obtained from *Dendropanax morbifera* Lev Are Dependent on the Collecting Season and Extraction Conditions. Food Sci. Biotechnol..

[15] Akram M., Kim K.A., Kim E.S., Syed A.S., Kim C.Y., Lee J.S., Bae O.N. (2016). Potent Anti-Inflammatory and Analgesic Actions of the Chloroform Extract of *Dendropanax morbifera* Mediated by the Nrf2/Ho-1 Pathway. Biol. Pharm. Bull..

[16] Birhanu B.T., Kim J.-Y., Hossain A., Choi J.-W., Lee S.-P., Park S.-C. (2018). An in Vivo Immunomodulatory and Anti-Inflammatory Study of Fermented *Dendropanax morbifera* Léveille Leaf Extract. BMC Complement. Altern. Med..

[17] Yoo D.-Y., Jung H.Y., Kwon H.J., Kim J.W., Nam S.M., Chung J.Y., Choi J.H., Kim D.W., Yoon Y.S., Hwang I.K. (2016). Effects of *Dendropanax morbifera* Léveille Extract on Hypothyroidism-Induced Oxidative Stress in the Rat Hippocampus. Food Sci. Biotechnol..

[18] Lee J.W., Kim K.S., An H.K., Kim C.H., Moon H.I., Lee Y.C. (2013). Dendropanoxide Induces Autophagy through Erk1/2 Activation in Mg-63 Human Osteosarcoma Cells and Autophagy Inhibition Enhances Dendropanoxide-Induced Apoptosis. PLoS ONE.

[19] Sachan R., Kundu A., Dey P., Son J.Y., Kim K.S., Lee D.E., Kim H.R., Park J.H., Lee S.H., Kim J.-H. (2020). *Dendropanax morbifera* Protects against Renal Fibrosis in Streptozotocin-Induced Diabetic Rats. Antioxidants.

[20] An N.Y., Kim J.E., Hwang D., Ryu H.K. (2014). Anti-Diabetic Effects of Aqueous and Ethanol Extract of *Dendropanax morbifera* Leveille in Streptozotocin-Induced Diabetes Model. J. Nutr. Health.

[21] Lee C., Yang M., Moon J.-O. (2019). Antioxidant and Hepatoprotective Effects of the Ethanol Extract of *Dendropanax morbifera* Leveille on the T-Butyl Hydroperoxide-Induced Hepg2 Cell Damages. Korean J. Pharmacogn..

[22] Bae D., Kim J., Lee S.-Y., Choi E.-J., Jung M.-A., Jeong C.S., Na J.-R., Kim J.-J., Kim S. (2015). Hepatoprotective Effects of Aqueous Extracts from Leaves of *Dendropanax morbifera* Leveille against Alcohol-Induced Hepatotoxicity in Rats and In Vitro Anti-Oxidant Effects. Food Sci. Biotechnol..

[23] Song J.H., Kwak S., Kim H., Jun W., Lee J., Yoon H.G., Choi K.C. (2019). *Dendropanax morbifera* Branch Water Extract Increases the Immunostimulatory Activity of Raw264. 7 Macrophages and Primary Mouse Splenocytes. J. Med. Food.

[24] Kim R.-W., Lee S.-Y., Kim S.-G., Heo Y.-R., Son M.-K. (2016). Antimicrobial, Antioxidant and Cytotoxic Activities of *Dendropanax morbifera* Léveille Extract for Mouthwash and Denture Cleaning Solution. J. Adv. Prosthodont..

[25] Chung I.-M., Kim M.-Y., Park S.-D., Park W.-H., Moon H.-I. (2009). In Vitro Evaluation of the Antiplasmodial Activity of *Dendropanax morbifera* against Chloroquine-Sensitive Strains of Plasmodium Falciparum. Phytother. Res..

[26] Yun J.-W., Kim S.-H., Kim Y.-S., Choi E.J., You J.-R., Cho E.-Y., Yoon J.-H., Kwon E., Kim H.-C., Jang J.-J. (2019). Preclinical Study of Safety of *Dendropanax morbifera* Leveille Leaf Extract: General and Genetic Toxicology. J. Ethnopharmacol..

[27] Chung I.-M., Seo S.-H., Kang E.-Y., Park S.-D., Park W.-H., Moon H.-I. (2009). Chemical Composition and Larvicidal Effects of Essential Oil of *Dendropanax morbifera* against *Aedes aegypti* L.. Biochem. Syst. Ecol..

[28] Lee S.Y., Choi E.J., Bae D.H., Lee D.W., Kim S. (2015). Effects of 1-Tetradecanol and Β-Sitosterol Isolated from *Dendropanax morbifera* Lev. On Skin Whitening, Moisturizing and Preventing Hair Loss. J. Soc. Cosmet. Sci. Korea.

[29] Shin D.C., Kim G.C., Song S.Y., Kim H.J., Yang J.C., Kim B. (2013). Antioxidant and Antiaging Activities of Complex Supercritical Fluid Extracts from *Dendropanax morbifera*, *Corni fructus* and *Lycii fructus*. Korea J. Herbol..

[30] Alvand Z.M., Rajabi H.R., Mirzaei A., Masoumiasl A. (2019). Ultrasonic and Microwave Assisted Extraction as Rapid and Efficient Techniques for Plant Mediated Synthesis of Quantum Dots: Green Synthesis, Characterization of Zinc Telluride and Comparison Study of Some Biological Activities. New J. Chem..

[31] Moon J.-Y., Ngoc L.T.N., Chae M., Van Tran V., Lee Y.-C. (2020). Effects of Microwave-Assisted *Opuntia humifusa* Extract in Inhibiting the Impacts of Particulate Matter on Human Keratinocyte Skin Cell. Antioxidants.

[32] Waterman P.G., Mole S. (1994). Analysis of Phenolic Plant Metabolites.

[33] Woisky R.G., Salatino A. (1998). Analysis of Propolis: Some Parameters and Procedures for Chemical Quality Control. J. Apic. Res..

[34] Mingle C.E., Newsome A.L. (2020). An Amended Potassium Persulfate Abts Antioxidant Assay Used for Medicinal Plant Extracts Revealed Variable Antioxidant Capacity Based Upon Plant Extraction Process. bioRxiv.

[35] Suganya P., Jeyaprakash K., Mallavarapu G.R., Murugan R. (2015). Comparison of the Chemical Composition, Tyrosinase Inhibitory and Anti-Inflammatory Activities of the Essential Oils of *Pogostemon plectranthoides* from India. Ind. Crops Prod..

[36] Tu P.T.B., Tawata S. (2015). Anti-Oxidant, Anti-Aging, and Anti-Melanogenic Properties of the Essential Oils from Two Varieties of *Alpinia zerumbet*. Molecules.

[37] Park C., Kim J., Hwang W., Lee B.D., Lee K. (2016). In Vitro Anti-Tyrosinase Activity of Viscumneoside III and Homoflavoyadorinin B Isolated from Korean Mistletoe (*Viscum album*). Korean J. Plant Resour..

[38] Casagrande R., Georgetti S.R., Verri W., Dorta D., Santos A.C., Fonseca M.J. (2006). Protective Effect of Topical Formulations Containing Quercetin against Uvb-Induced Oxidative Stress in Hairless Mice. J. Photochem. Photobiol. B Biol..

[39] Fulop T., Khalil A., Larbi A. (2012). The Role of Elastin Peptides in Modulating the Immune Response in Aging and Age-Related Diseases. Pathol. Biol..

[40] Thring T.S., Hili P., Naughton D.P. (2009). Anti-Collagenase, Anti-Elastase and Anti-Oxidant Activities of Extracts from 21 Plants. BMC Complement. Altern. Med..

[41] Horng C.-T., Wu H.-C., Chiang N.-N., Lee C.-F., Huang Y.-S., Wang H.-Y., Yang J.-S., Chen F.-A. (2017). Inhibitory Effect of Burdock Leaves on Elastase and Tyrosinase Activity. Exp. Ther. Med..

[42] Popoola O.K., Marnewick J.L., Rautenbach F., Ameer F., Iwuoha E.I., Hussein A.A. (2015). Inhibition of Oxidative Stress and Skin Aging-Related Enzymes by Prenylated Chalcones and Other Flavonoids from *Helichrysum teretifolium*. Molecules.

[43] Villareal M.O., Kume S., Neffati M., Isoda H. (2017). Upregulation of Mitf by Phenolic Compounds-Rich *Cymbopogon schoenanthus* Treatment Promotes Melanogenesis in B16 Melanoma Cells and Human Epidermal Melanocytes. BioMed Res. Int..

[44] Lin Y.-S., Chen H.-J., Huang J.-P., Lee P.-C., Tsai C.-R., Hsu T.-F., Huang W.-Y. (2017). Kinetics of Tyrosinase Inhibitory Activity Using *Vitis vinifera* Leaf Extracts. BioMed Res. Int..

[45] Kim Y.-J., Uyama H., Kobayashi S. (2004). Inhibition Effects of (+)-Catechin–Aldehyde Polycondensates on Proteinases Causing Proteolytic Degradation of Extracellular Matrix. Biochem. Biophys. Res. Commun..

[46] Baylac S., Racine P. (2004). Inhibition of Human Leukocyte Elastase by Natural Fragrant Extracts of Aromatic Plants. Int. J. Aromather..

