# Supplementary material for: Microwave-Assisted Dendropanax morbifera Extract for Cosmetic Applications
Source: Antioxidants (Basel). 2022 May 19;11(5):998. doi: 10.3390/antiox11050998 (PMC9137482; doi:10.3390/antiox11050998)
Supplement: Supplementary file 1 [file antioxidants-11-00998-s001.zip › antioxidants-1719869-supplementary.pdf]

Supplementary Materials

Table S1. List of samples and the absorbance measurement results for evaluating phenolic compounds.

Concentration (µg/mL)	Absorbance
GA 50 µg/mL	0.0281
GA 100 µg/mL	0.0758
GA 200 µg/mL	0.1946
GA 400 µg/mL	0.4346
GA 500 µg/mL	0.5189
Black extract 5%	0.2526
Transparent extract 5%	0.0043
MA-DME	0.3207

Table S2. List of samples and the absorbance measurement results for evaluating flavonoid compounds.

Concentration (µg/mL)	Absorbance
Quercetin 5 µg/mL	0.1714
Quercetin 10 µg/mL	0.3599
Quercetin 15 µg/mL	0.5457
Quercetin 30 µg/mL	1.0018
Quercetin 40 µg/mL	1.2665
Quercetin 50 µg/mL	1.4711
Black extract 5%	0.6769
Transparent extract 5%	0.0053
MA-DME	1.0117

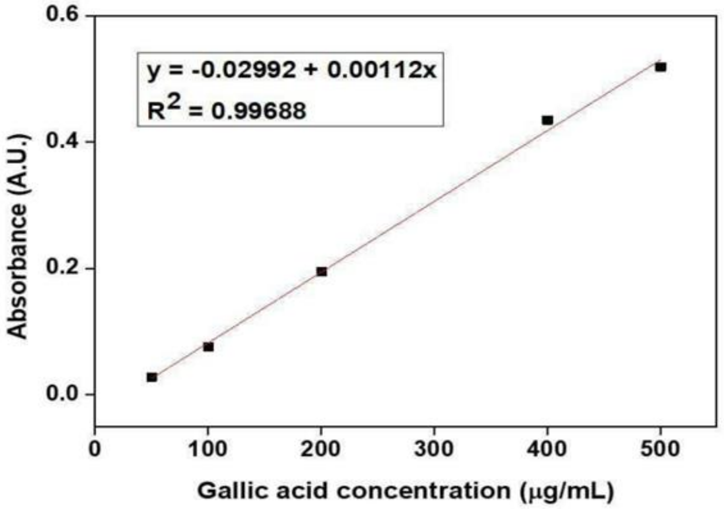

Figure S1. The calibration curve for quantification of phenolic compounds.

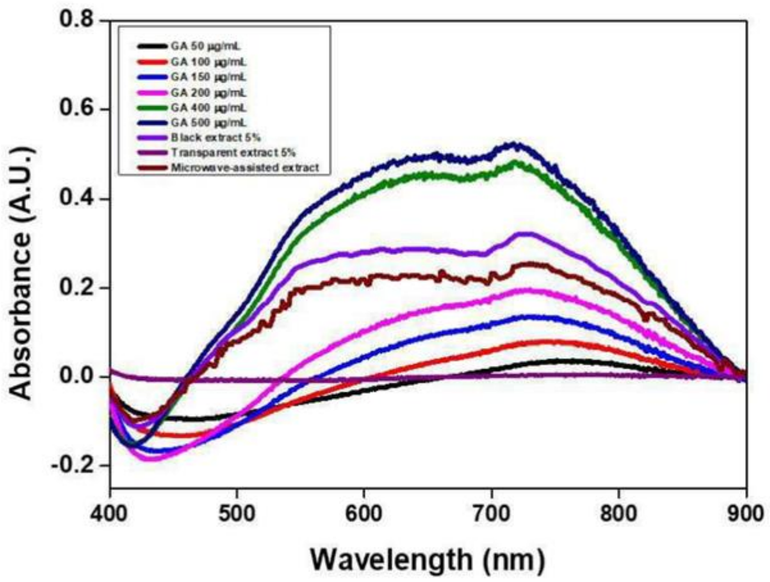

Figure S2. The absorbance measurement results to evaluate the phenolic compounds.

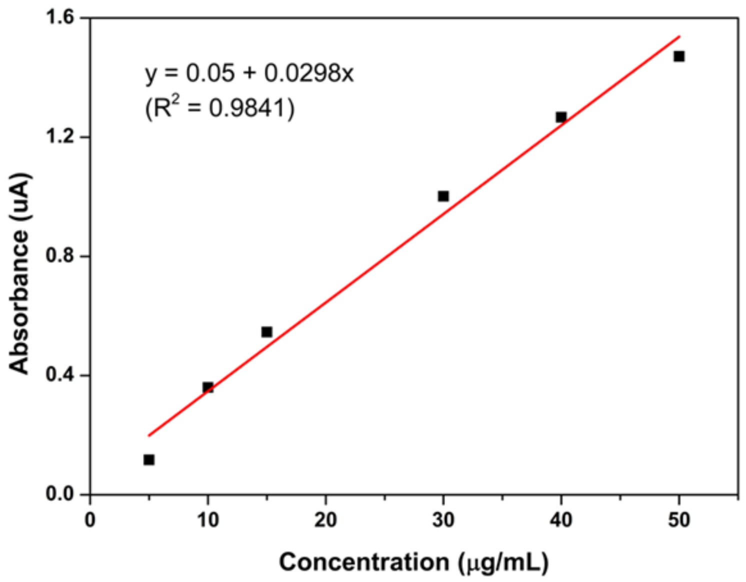

Figure S3. The calibration curve for quantification of flavonoid compounds.

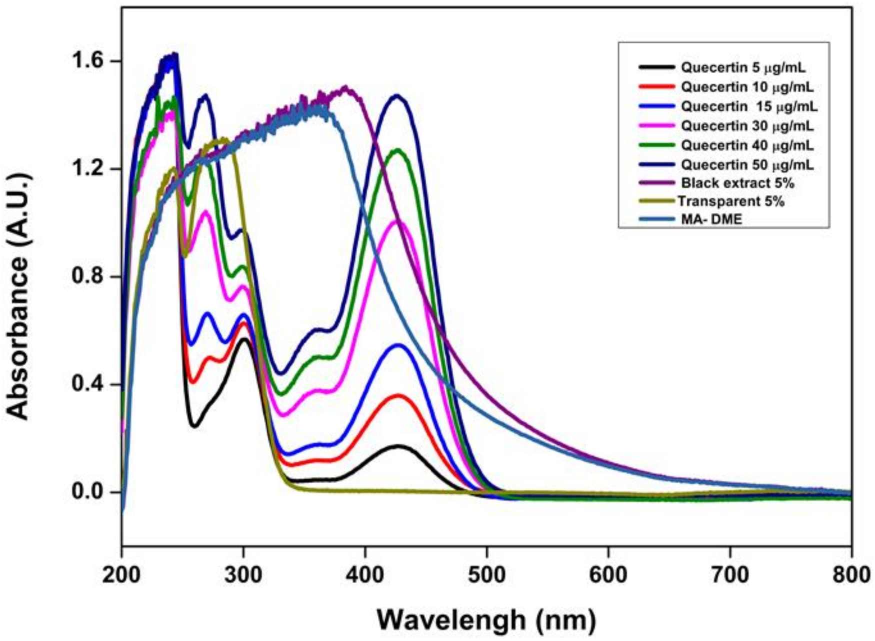

Figure S4. The absorbance measurement results to evaluate the flavonoid compounds.
